# Modifications of Erectile Tissue Components in the Penis during the Fetal Period

**DOI:** 10.1371/journal.pone.0106409

**Published:** 2014-08-29

**Authors:** Carla B. M. Gallo, Waldemar S. Costa, Angelica Furriel, Ana L. Bastos, Francisco J. B. Sampaio

**Affiliations:** 1 Urogenital Research Unit, State University of Rio de Janeiro, UERJ, Rio de Janeiro, Brazil; 2 Department of Morphology, Fluminense Federal University, Rio de Janeiro, Brazil; Semmelweis University, Hungary

## Abstract

**Background:**

The penile erectile tissue has a complex microscopic anatomy with important functions in the mechanism of penile erection. The knowledge of such structures is necessary for understanding the normal physiology of the adult penis. Therefore, it is important to know the changes of these penile structures during fetal development. This study aims to analyze the development of the main components of the erectile tissue, such as collagen, smooth muscle fibers and elastic system fibers, in human fetuses.

**Methodology/Principal Findings:**

We studied the penises of 56 human fetuses aged 13 to 36 weeks post-conception (WPC). We used histochemical and immunohistochemical staining, as well as morphometric techniques to analyze the collagen, smooth muscle fibers and elastic system fibers in the corpus cavernosum and in the corpus spongiosum. These elements were identified and quantified as percentage by using the Image J software (NIH, Bethesda, USA). From 13 to 36 WPC, in the corpus cavernosum, the amount of collagen, smooth muscle fibers and elastic system fibers varied from 19.88% to 36.60%, from 4.39% to 29.76% and from 1.91% to 8.92%, respectively. In the corpus spongiosum, the amount of collagen, smooth muscle fibers and elastic system fibers varied from 34.65% to 45.89%, from 0.60% to 11.90% and from 3.22% to 11.93%, respectively.

**Conclusions:**

We found strong correlation between the elements analyzed with fetal age, both in corpus cavernosum and corpus spongiosum. The growth rate of these elements was more intense during the second trimester (13 to 24 WPC) of gestation, both in corpus cavernosum and in corpus spongiosum. There is greater proportional amount of collagen in the corpus spongiosum than in corpus cavernosum during all fetal period. In the corpus spongiosum, there is about four times more collagen than smooth muscle fibers and elastic system fibers, during all fetal period studied.

## Introduction

While formed by only 2 types of non-stromal cells; smooth muscle cells and endothelial cells, the erectile tissue of the penis (corpus cavernosum and corpus spongiosum) has a complex microscopic anatomy, forming a network of sinusoidal spaces. The erectile tissue of the human penis is composed of elastic fibers, collagen fibers, smooth muscles, arteries and veins, and has important functions in the mechanism of penile erection [1]–[6]. The trabeculae of the erectile tissue are major penile structures involved in erection and are formed by the extracellular matrix and smooth muscle fibers. These elements give support to nerves, sinusoids, arteries and endothelium [6], [7]. Histochemical and immunohistochemical analyses, of which some were associated with morphometry, have characterized structural components in the erectile tissue of adult penis [3], [4], [8] and also, in preliminary works [4], [5], these techniques have been used to investigate erectile tissue in human fetal penis.

The knowledge of such structures is necessary for understanding the normal physiology of the adult penis, commonly altered in different clinical or experimental situations [9]–[11]. Therefore, it is important to know the changes of these penile structures during fetal development.

Recently, it has been demonstrated the development of the area of the penis and corpora cavernosa and corpus spongiosum during the fetal period [12]. Nevertheless, until now, the morphology, development, modifications and distribution of the erectile tissue in the fetal penis is unknown.

Therefore, the objective of the present work is to analyze, qualitatively and quantitatively, in the corpora cavernosa (CC) and corpus spongiosum (CS), the development of the main components of the penile erectile tissue, such as collagen, smooth muscle fibers and elastic system fibers, during the whole fetal period (13 to 36 weeks post-conception – WPC), providing normative patterns of growth.

## Materials and Methods

This research was performed at Urogenital Research Unit, State University of Rio de Janeiro. The Ethics Committee of Pedro Ernesto University Hospital (State University of Rio de Janeiro) approved the project and waived the need of informed consent.

We studied 56 penises obtained from normal human fetuses that had died of causes unrelated to the urogenital tract. The fetuses were from the Department of Anatomy of the State University of Rio de Janeiro collection. All subjects were well preserved and none of them had any kind of detectable congenital malformation. The gestational age of the fetuses ranged from 13 to 36 weeks post-conception (corresponding to 15 to 38 menstrual weeks), and was estimated by the greatest foot length method [13]–[15]. We used 1 to 5 fetuses in each gestational age.

### Tissue Sample Preparation

After dissection, the penis was removed, cross-sectioned at its mid-shaft and immediately immersed in 10% formalin phosphate buffer solution for 24 hours. Afterwards, the samples were embedded in paraffin and sectioned with 5-µm at intervals of 200-µm between each section.

The samples for histological analysis were obtained from the mid-shaft of the penis. All specimens were processed identically to minimize potential differences caused by processing. The sections were stained with Masson's trichrome for quantification of collagen, immunostaining for elastin for quantification of elastic system fibers and immunostaining for alpha-actin for quantification of smooth muscle fibers.

### Morphometric Analysis

All sections were photographed under the same conditions with a resolution of 2040×1536 pixels, with a digital camera coupled to a microscope and stored in a TIFF format file.

We obtained 5 sections from each penis and we analyzed 5 fields on each section and we performed 3 measurements for each field; therefore, we obtained 75 measurements for each penis, for each element analyzed. The result was the mean of percentages obtained for each color range. All quantifications were performed with a final magnification of X1000. Both for corpus cavernosum and for corpus spongiosum, the quantifications were carried out in the same manner.

**Figure 1:**
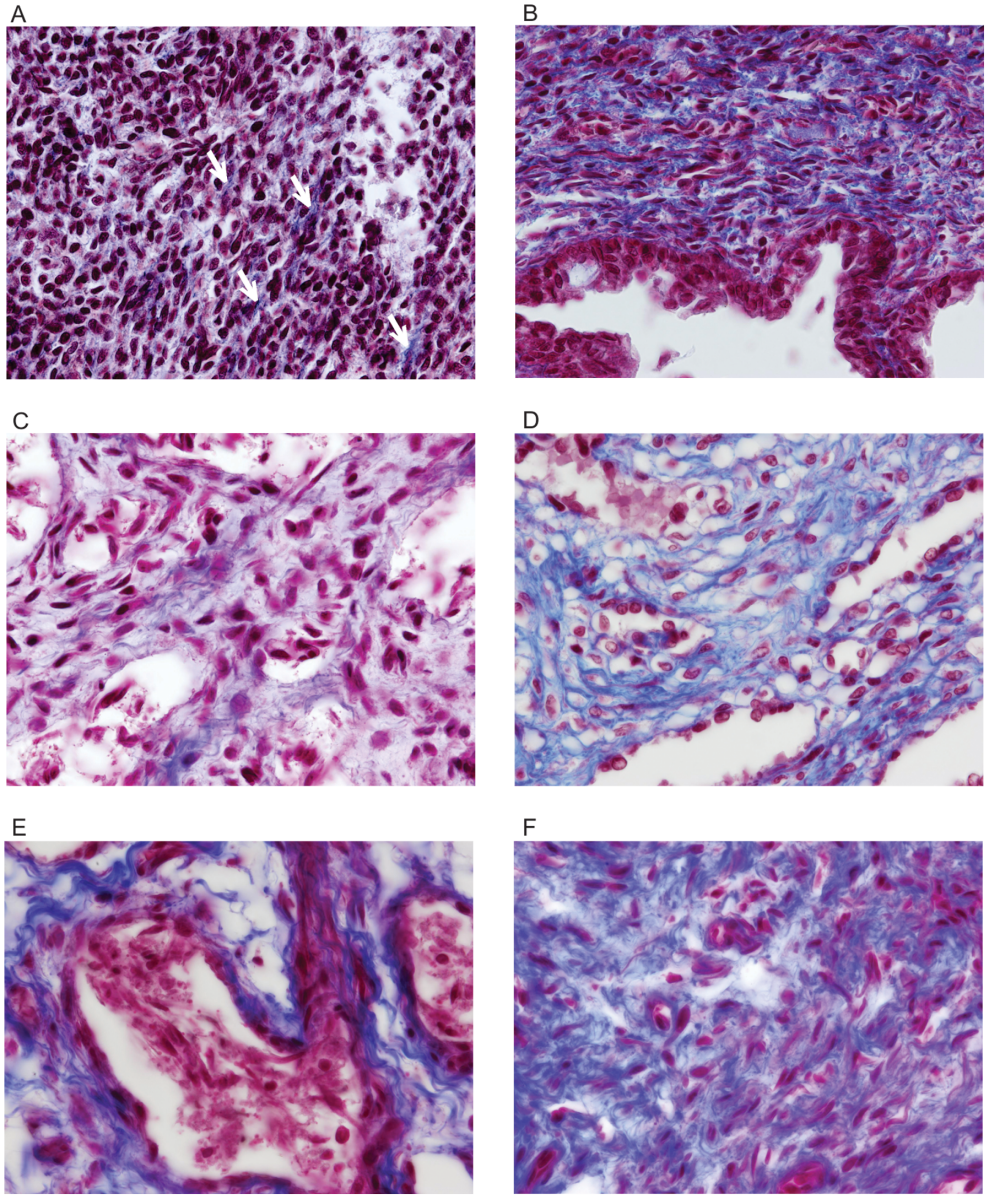
Photomicrographs of collagen in the corpus cavernosum and corpus spongiosum (blue color) of fetal penis at different ages in weeks post-conception (WPC). A) Corpus cavernosum, fetus of 13 WPC – arrows indicate small amount of collagen; B) Corpus spongiosum, fetus of 13 WPC; C) Corpus cavernosum, fetus of 22 WPC; D) Corpus spongiosum, fetus of 21 WPC; E) Corpus cavernosum, fetus of 32 WPC; F) Corpus spongiosum, fetus of 32 WPC. Masson's trichrome, X1000.

The Image J software 1.46r version (NIH, Bethesda, MD, USA), loaded with its own “plugin” (http://www.imagej.nih.gov/ij) has been used for morphometrical analyses. The percentage of collagen, smooth muscle fibers and elastic system fibers, was performed by using the plugin Color Segmentation. With the image in the software (Figure 1), by using the tool Point-Cross toolbar, the selection is performed by RGB color ranges and we can control the standard deviation. After color selection is performed, each color is detected and, consequently, the percentage amount expressed.

### Statistical Analysis

We performed the statistical analysis using the mean values for each element (collagen, smooth muscle and elastic fibers) by simple linear regression assessing the association between the variables analyzed with the fetal age. In addition, the correlation coefficient (r) and the p value were obtained for each regression analysis. P≤0.05 was considered to indicate statistical significance. Software Graphpad Prism 5.0 was used.

## Results

### Collagen

During the fetal period in humans (13 to 36 weeks post-conception – WPC), the collagen accounted from 19.88% to 36.60% in the corpus cavernosum of the penis and from 34.65 to 45.89% in the corpus spongiosum (Figure 1).

### Smooth Muscle Fibers

**Figure 2:**
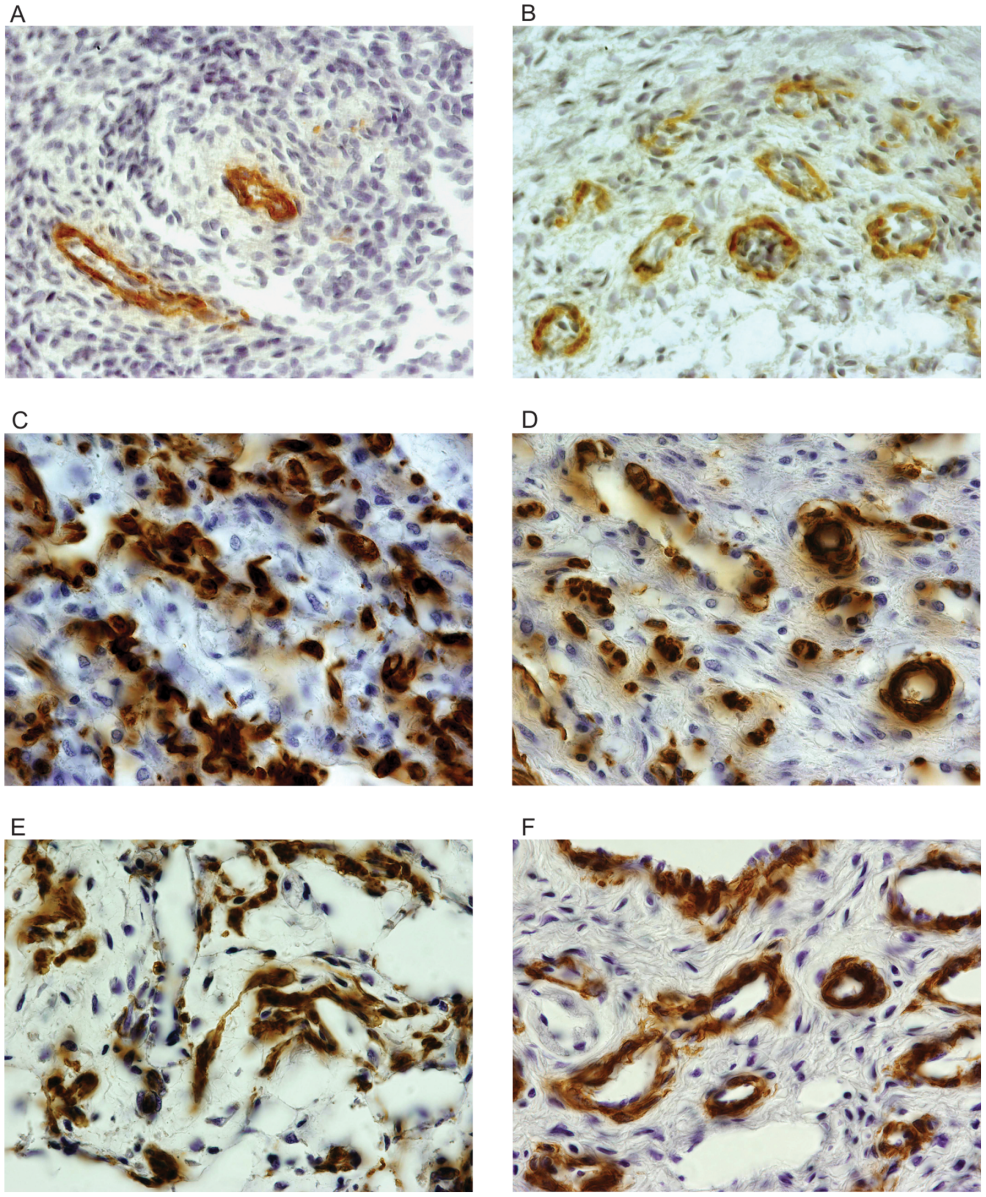
Photomicrographs of smooth muscle fibers in the corpus cavernosum and corpus spongiosum (brownish color) of fetal penis at different ages in weeks post-conception (WPC). A) Corpus cavernosum, fetus of 13 WPC; B) Corpus spongiosum, fetus of 13 WPC; C) Corpus cavernosum, fetus of 21 WPC; D) Corpus spongiosum, fetus of 21 WPC; E) Corpus cavernosum, with 30 WPC fetus; F) Corpus spongiosum, fetus of 30 WPC. Immunostaining for alpha actin, X1000.

The smooth muscle fibers accounted from 4.39% to 29.76% in the corpus cavernosum of the penis and from 0.60% to 11.90% in the corpus spongiosum (Figure 2), from 13 to 36 WPC.

### Elastic System Fibers

**Figure 3:**
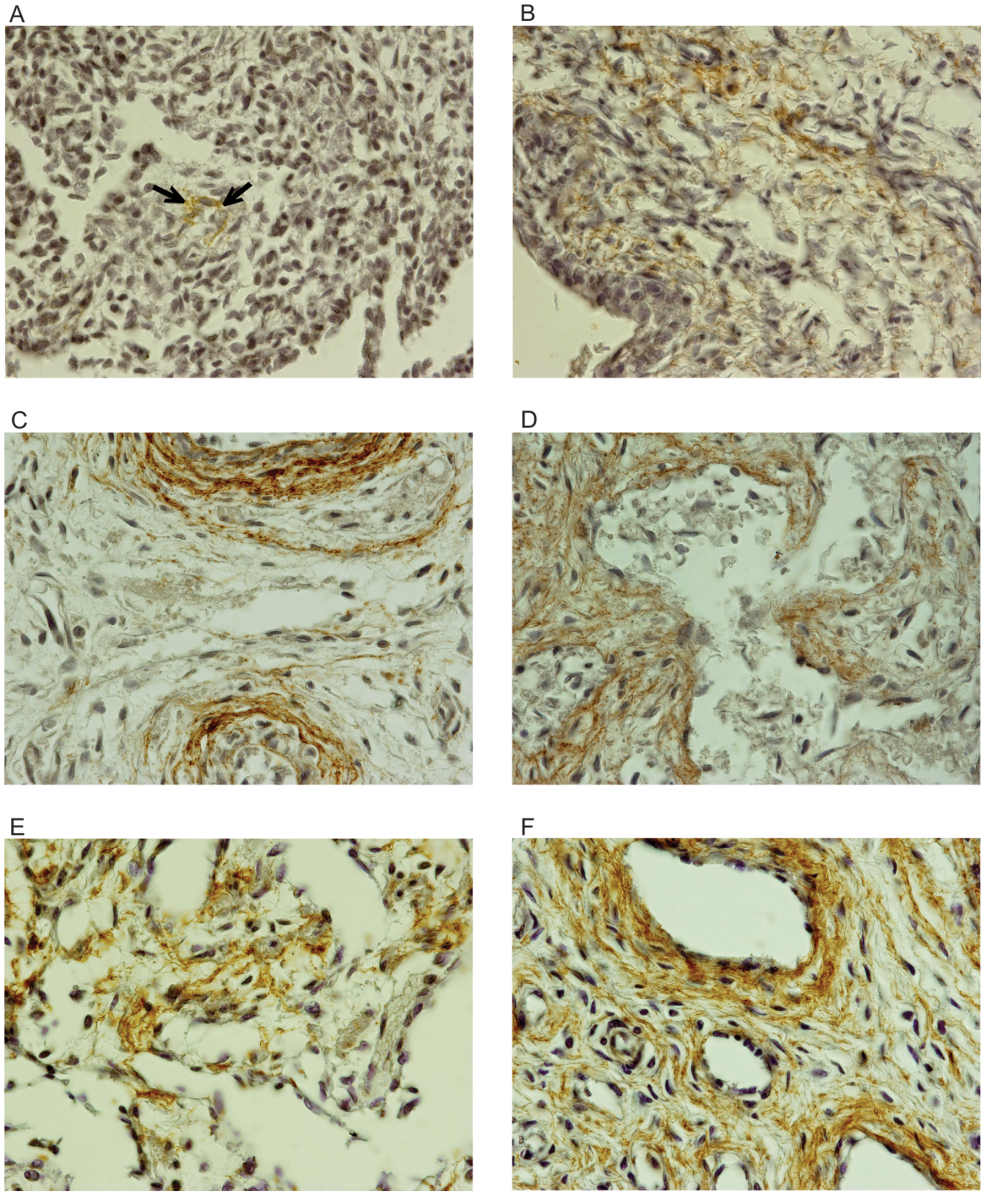
Photomicrographs of elastic system fibers in the corpus cavernosum and corpus spongiosum (brown tint) of fetal penis at different ages in weeks post-conception (WPC). A) Corpus cavernosum, fetus of 13 WPC; B) Corpus spongiosum, fetus of 13 WPC; C) Corpus cavernosum, fetus of 20 WPC; D) Corpus spongiosum, fetus of 20 WPC; E) Corpus cavernosum, fetus of 30 WPC; F) Corpus spongiosum, fetus of 30 WPC. Immunostaining for elastin, X1000.

During the fetal period in humans (13 to 36 WPC), the elastic system fibers accounted from 1.91% to 8.92% in the corpus cavernosum and from 3.22% to 11.93% in the corpus spongiosum (Figure 3).

The linear regression analysis indicated that collagen, smooth muscle fibers and elastic system fibers concentration, correlated significantly and positively with fetal age, during the whole fetal period studied (13 to 36 WPC), and also during the second trimester (13 to 24 WPC) and during the third trimester (25 to 36 WPC) of gestation, when analyzed separately, both in corpus cavernosum and in corpus spongiosum. In addition, the linear regression analysis demonstrated a more intense growth rate of collagen, smooth muscle and elastic system fibers, during the second trimester of gestation, when compared to the third trimester, both in corpus cavernosum and in corpus spongiosum.

**Figure 4:**
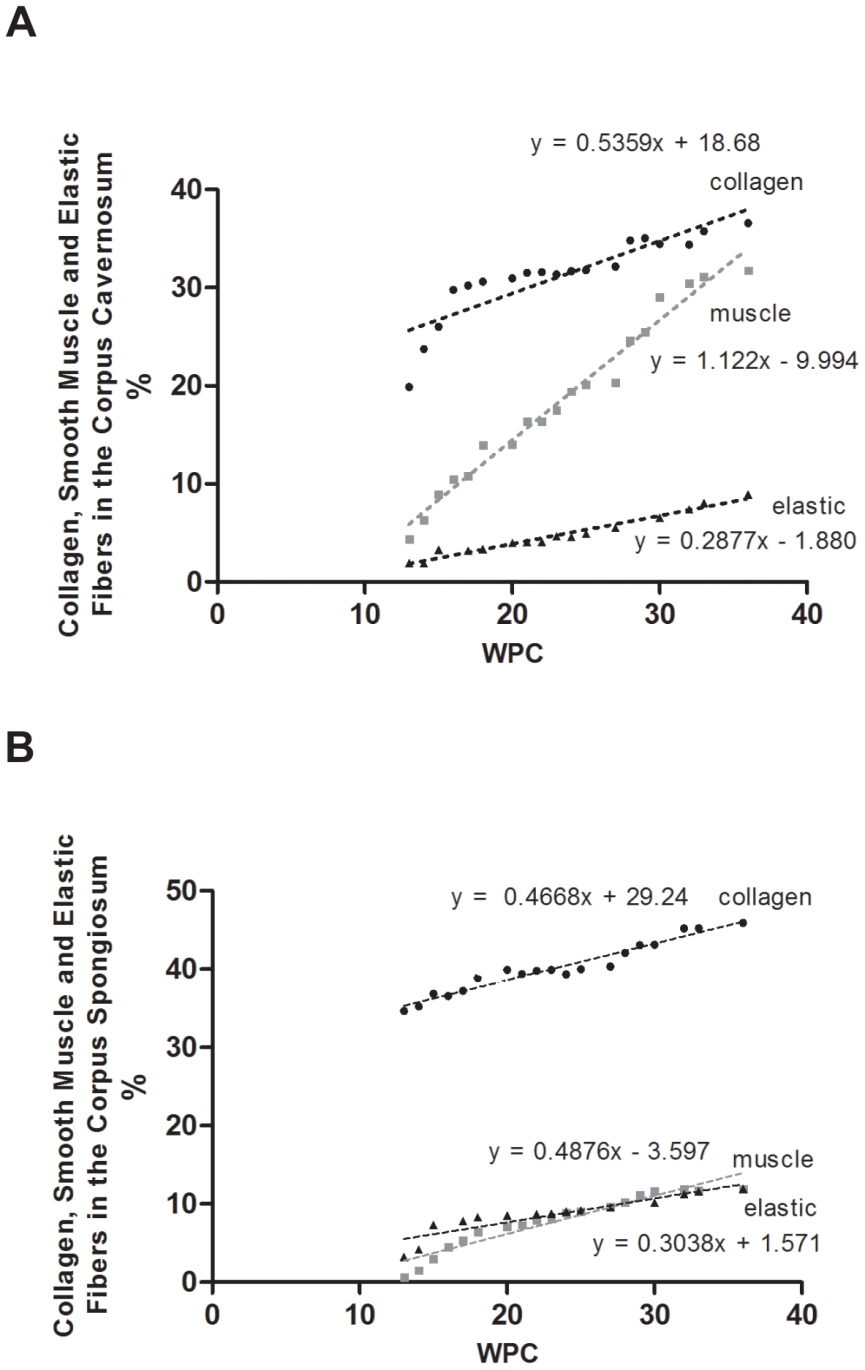
Correlation between collagen, smooth muscle fibers and elastic system fibers, in percentage, with fetal age, during the fetal period studied (13 to 36 weeks post-conception – WPC). The points plotted represent the mean values obtained for each week studied. **A**) Analysis in the corpus cavernosum. Linear regression indicated that the growth is correlated significantly and positively, with fetal age (collagen: r^2^ = 0.9771, p<0.0001; smooth muscle: r^2^ = 0.9516, p<0.0001; elastic fibers: r^2^ = 0.9122, p<0.0001). **B**) Analysis in the corpus spongiosum. Linear regression indicated that the growth is correlated significantly and positively, with fetal age (collagen: r^2^ = 0.9464, p<0.0001; smooth muscle: r^2^ = 0.9153, p<0.0001; elastic fibers: r^2^ = 0.8299, p<0.0001).

Figure 4 shows the correlation between collagen, muscle fibers and elastic system fibers, in percentage, in the corpus cavernosum and corpus spongiosum, with fetal age, during the fetal period studied (13 to 36 WPC).

## Discussion

Previous studies from our group, in various organs of the urogenital system, including the penis, over the past 25 years, showed the importance of establishing growth patterns in normal human fetuses [4], [5], [16]–[18]. In addition, we studied the urogenital system in fetuses with congenital anomalies, as prune-belly syndrome and anencephaly, and compared these results with normal fetuses, with the view to possible use of anencephalic, for example, as potential donors of tissues and organs [19]–[21].

In the present work, we were able to include fetuses that covered almost all fetal period, 13 to 36 weeks post-conception, which corresponds from 15 to 38 menstrual weeks. Concerning the penis, this kind of study is inexistent in the literature and is very important, because the transformations undergone by the organs during fetal development are very fast [22].

With reference to penis development during the fetal period, the only studies available in the literature, apart from that of our group [5], [6], [17]–[21], [23] were performed by ultrasound or other imaging methods in utero, and were related only to penile length [24]–[27].

In this study, we confirmed that at the 13th week post-conception (WPC), the corpora cavernosa and the corpus spongiosum, as well as the septa intra-cavernous, are already present as well individualized anatomical structures.

After analyzing the penile erectile tissue structures, with respect to collagen, it was observed that at the 13th WPC, in the corpus cavernosum, there is a predominance of mesenchymal tissue and abundance of nuclei with small amount of incipient collagen (Figure 1A), unlike the corpus spongiosum, where at the 13th WPC it is found an appreciable amount of collagen (Figure 1B and 4). The quantitative analysis confirms these observations and this difference is maintained during all fetal period (Figure 4).

As gestational age increases, the collagen, both in the corpus cavernosum and corpus spongiosum, becomes more evident and increases in quantity (Figure 1). The quantitative analysis shows objectively and confirms these results (Figure 4). By linear regression analysis, comparing the growth of collagen (both in the corpus cavernosum and corpus spongiosum), between the second and third trimester, we observed that collagen increases with a more intense rhythm in the second trimester.

A previous study from our laboratory [5] analyzing five normal fetuses at 28 WPC had already demonstrated that collagen is an important and well-differentiated component of the corpus cavernosum in the third trimester of gestation. Another previous study from our laboratory [23], with normal human fetuses, infants, children and adults, using biochemical methods, showed that the total concentration of collagen in the fetal penis increased progressively, and almost doubled between the 17th and 33rd WPC, suggesting that major changes in the extracellular matrix of the penis occur in this gestational period. Also, in a previous study from our laboratory, Costa et al. [8] showed that the percentage of collagen in the corpus cavernosum is around 40% in normal adults. This percentage is around 3% above the average of 36.6% that we have found for fetuses in late pregnancy (36th WPC, Figure 4); and this demonstrated that the percentage of collagen in the corpus cavernosum increases slightly after birth.

From the 22nd WPC, one begins to observe in the corpus cavernosum, a relationship between smooth muscle and collagen, evidencing the proportional increase of the smooth muscle, unlike what happens in the corpus spongiosum, where the muscle remains in small amount (Figures 1 and 2). The linear regression analysis, comparing the proportion of collagen and smooth muscle fibers in corpus cavernosum and corpus spongiosum, objectively confirmed these observations (Figure 4). The linear regression analysis also showed that this difference is significant and is more pronounced in the third trimester of gestation.

Still in the corpus cavernosum, as the fetus grows, it is noted that collagen increases progressively and in parallel, there is an increase of muscle fibers and elastic system fibers (Figures 1 to 3). The quantification and the linear regression analysis showed objectively, however, that the increase in smooth muscle fibers is proportionally greater in the corpus cavernosum considering all fetal period (Figure 4). The linear regression analysis showed that the smooth muscle growth is strongly correlated with the fetal age and is more intense during the second trimester of gestation. Also, the quantitative analysis demonstrated a growth with predominance of smooth muscle in the trabeculae and a proportional decrease of collagen (Figure 2).

A previous study from our laboratory [4], analyzing 7 fetuses at 24th WPC found a distribution of smooth muscle cells in corpus cavernosum ranging from 17.52% to 27.76% with an average of 22.72%, which is in accordance with the present work. Also, a previous study from our lab [8] demonstrated that the percentage of smooth muscle fibers in the corpus cavernosum is around 40% in normal adults. This is approximately 26% more than the mean of 29.76% that we have found for fetuses in late pregnancy (36 WPC, Figure 4), showing that the proportional growth of smooth muscle fibers continues after birth.

These findings, concerning smooth muscle in the corpus cavernosum, are unique when we compare with other mammals, such as rats, in which the corpora cavernosa is predominantly composed of collagen [28], or rabbit, where there is an abundance of elastic fibers [29], or the wild boar, where smooth muscle fibers are scarce in the corpus cavernosum [30].

Regarding the corpus spongiosum, the linear regression analysis also showed that the smooth muscle fibers grow with a more intense rhythm in the second trimester of gestation. Still in the corpus spongiosum, as the fetus grows, it is noted that collagen increases progressively, and, in parallel, there is an increase of smooth muscle fibers and elastic fibers, although fewer and less intense, when compared to corpus cavernosum (Figures 1 to 4). The quantitative analysis shows objectively, however, that collagen increase is proportionately larger, considering the area of the corpus spongiosum during the fetal period. The quantitative analysis also demonstrated that there is about 4 times more collagen than smooth muscle fibers and elastic system fibers in the corpus spongiosum throughout the fetal period. Also, in the corpus spongiosum, it is observed that the amount of muscle fibers and elastic fibers are equivalent (Figure 4).

With respect to elastic system fibers, we verified that with increasing of gestational age, the fibers become more apparent and increase in quantity both in corpus cavernosum and in the corpus spongiosum (Figure 3). The linear regression analysis of the quantitative data (Figure 4) confirms these findings objectively. In addition, the linear regression analysis showed that elastic fibers grow in a more intense rhythm in the corpus cavernosum during the second trimester, while in the corpus spongiosum the growth rate is similar along all fetal period studied. Throughout the fetal period, the amount of elastic system fibers is greater in the corpus spongiosum when compared to corpus cavernosum (Figure 4). A previous study from our group [22] with 10 fetuses between 15 and 36 WPC showed that the concentration of elastic fibers in the spongy urethra increases significantly with fetal age and the concentration varied from 5 to 14%, which is consistent with the results of this study. Also, in a previous study from our laboratory, Costa et al. [9] showed that the percentage of elastic system fibers is about 13% in normal adults. This amount is very similar to the mean of 11.93% that we found in the corpus cavernosum of fetuses, later in gestation (36th WPC, Figure 4), and demonstrates that the percentage of elastic system fibers increases slightly after birth.

## Conclusions

We found strong correlation between collagen, smooth muscle fibers and elastic system fibers growth with the fetal age, both in corpus cavernosum and in corpus spongiosum. The growth rate of all elements analyzed was more intense during the second trimester (13 to 24 WPC) of gestation when compared to the third trimester (25 to 36 WPC), both in corpus cavernosum and in corpus spongiosum.

There is greater proportional amount of collagen in the corpus spongiosum than in corpus cavernosum during all fetal period. With increasing gestational age, in the corpus cavernosum occurs a growth of smooth muscle predominantly in the trabeculae with a proportional decrease of collagen.

The elastic system fibers are the constituent elements in lesser amounts in the erectile tissue of the corpus cavernosum throughout the fetal period. In the corpus spongiosum, there is about four times more collagen than smooth muscle fibers and elastic system fibers, during all fetal period studied. In the corpus spongiosum, along the fetal period, the percentage of smooth muscle fibers and elastic system fibers are equivalent.
